# Effects of mitotane treatment on human steroid metabolism: implications for patient management

**DOI:** 10.1530/EC-12-0028

**Published:** 2012-07-21

**Authors:** L Ghataore, I Chakraborti, S J Aylwin, K-M Schulte, D Dworakowska, P Coskeran, N F Taylor

**Affiliations:** 1 Department of Clinical Biochemistry King's College Hospital London, SE5 9RS UK; 2 Department of Medicine King's College Hospital London, SE5 9RS UK; 3 Department of Endocrinology and Internal Medicine Medical University of Gdansk Gdansk Poland

**Keywords:** steroid, cortisol, mitotane, GC–MS

## Abstract

Mitotane (o,p'-DDD), an oral adrenolytic agent for treatment of advanced adrenocortical carcinoma (ACC), is reported to inhibit cortisol biosynthesis *in vitro* and enhance production from exogenous cortisol of urinary 6β-hydroxycortisol and unidentified polar unconjugated metabolites. We examined urinary steroid profiles by gas chromatography–mass spectrometry of patients with histologically confirmed ACC following surgery, receiving a) hydrocortisone alone (three males and three females) and b) mitotane and hydrocortisone (six males and 11 females). Samples were collected after plasma mitotane had reached the therapeutic range of 14–20 mg/l. Increased excretion of polar unconjugated steroids during mitotane treatment was confirmed, with 6β-hydroxycortisol and 6β-hydroxy-20-dihydrocortisols predominating. The proportion of additionally hydroxylated metabolites was <2% in untreated controls and 52, 35–52% (mean, range) in the mitotane plus hydrocortisone group. Ratios of 5α-/5β- and 20β-/20α-metabolites of administered cortisol were decreased 50-, 15-fold, and 14-, 8-fold respectively (males, females – mean values) but with no change in metabolite ratios that reflect oxidoreduction at C11 or C20. Patterns of decrease in 5α- relative to 5β-reduced metabolites were similar to those of patients with 5α-reductase 2 deficiency or on treatment with the 5α-reductase 2 inhibitor finasteride but different from those of patients on dutasteride, indicating specific inhibition of 5α-reductase 2. We conclude that mitotane causes consistent changes in cortisol catabolism, most of which have not been previously recognised. These need not interfere with early detection of ACC recurrence. Induction of 6β-hydroxylation offers an explanation for a reported decrease in cortisol bioavailability. Mitotane also has potential as a unique steroid metabolic probe for 20β-reduction.

## Introduction

Mitotane (o,p'-DDD) plays an important role in adjuvant therapy of adrenocortical carcinoma (ACC) and in advanced stage disease (1). This application was first reported in 1959 by Bergenstal *et al*. (2), but mitotane has only been in widespread use since publication in 2007 of the large retrospective study of Terzolo *et al*. (3). Accumulated knowledge of the mode of action of mitotane together with its effects on steroid synthesis and catabolism is patchy.

Mitotane targets adrenocortical tissue but its precise mode of action is not fully understood. It requires metabolic activation via hydroxylation at the β carbon (C2) followed by dehydrochlorination to form an acyl chloride (4). This binds covalently to mitochondrial proteins (4), causing selective adrenal toxicity via mitochondrial destruction (5). Marked disruption of the zona fasciculata and reticularis but not glomerulosa has been shown in dogs (6). Cytotoxicity is also due to oxidative damage via free radical production (reviewed by Schteingart (7)). Dogs are the species most sensitive to mitotane. Human adrenal mitochondria have much lower capacity to both activate mitotane and bind its products and are thus less responsive (4).

Mitotane inhibits adrenocortical mitochondrial cholesterol side chain cleavage (CYP11A1) and 11β-hydroxylation (CYP11B1) activities in bovine adrenal tissue (8). Inhibition of CYP11B1 and 18-hydroxylation (CYP11B2) activities has been shown in slices of adrenal glands taken from patients treated with mitotane (9).

Patients with Cushing's syndrome receiving mitotane have shown decrease in the adrenal cortisol secretion rate over time but a faster clinical benefit, suggesting that cortisol bioavailability is also diminished (10, 11). This has been attributed to hepatic microsomal enzyme induction (8, 12) but the cortisol metabolic clearance rate, as estimated by classical techniques, is reported to be unchanged (10, 13). An increased dose requirement for hydrocortisone replacement during mitotane treatment is well established in clinical practice. Cortisol monitoring by standard methods is unhelpful due to mitotane-induced cortisol binding globulin increase and ACTH is more useful, with addition of free cortisol assay when patients have continuing Cushing's syndrome (1).

**Figure 1:**
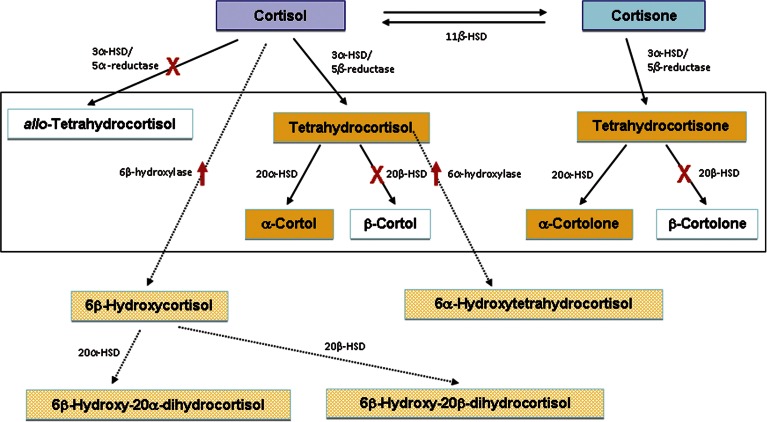
Metabolic chart showing generation of the major cortisol metabolites (boxed) in the human, together with the major changes brought about by mitotane. Crosses show pathways inhibited by mitotane and upward arrows show pathways stimulated by mitotane. Names shaded white are for steroids that show decreases after mitotane.

Normal human cortisol catabolism takes place primarily in the liver, with excretion into urine as glucuronide conjugates. These comprise (see box in Fig. 1) A-ring-reduced products 5β-tetrahydrocortisone and 5α- and 5β-tetrahydrocortisol; further reduction at C20 gives rise to the 20α- and 20β-hydroxy products, cortolones and cortols. Cortisol and 6β-hydroxycortisol are mainly present in unconjugated form and together comprise <2% of the total products derived from cortisol.

The only reports of mitotane effects on extra-adrenal cortisol metabolism (10, 11, 13) note a rapid change in metabolism of administered cortisol after starting treatment, comprising a decrease in urinary tetrahydrocortisone, 5α- and 5β-tetrahydrocortisol and a corresponding increase in excretion in an unconjugated polar steroid fraction, with 6β-hydroxycortisol as the only identified component.

We report here the results of urinary steroid profiling using a high-resolution capillary column for monitoring patients after removal of an ACC who were receiving mitotane combined with hydrocortisone (cortisol). We consistently found profound decreases in the common cortisol metabolites that are 5α- or 20β-reduced and an increased excretion of several polar unconjugated cortisol metabolites. As hydrocortisone treatment is known to independently result in changes in urinary cortisol metabolite proportions compared with those derived from endogenous production, we took account of these by including a group of patients after removal of an ACC receiving only hydrocortisone, in addition to comparing with control data obtained from healthy volunteers. In order to seek to identify which 5α-reductase isoform was being targeted by mitotane, we also made comparisons with steroid excretion in patients with 5α-reductase 2 deficiency (5ARD2) and those receiving the 5α-reductase inhibitors finasteride or dutasteride.

Our findings of increase in polar cortisol metabolites further explain the observed increase in dose requirement in patients receiving hydrocortisone, while 5ARD2 inhibition would be predicted to diminish dihydrotestosterone generation from testosterone, so that hypogonadal males might require additional androgen supplementation. Urinary steroid profile results obtained during monitoring for identified markers of ACC activity in patients after surgery can be appropriately interpreted in the light of this new knowledge.

## Subjects and methods

### Patients

The urine steroid data presented were obtained from samples submitted to our service for clinical purposes. Study groups comprised patients after surgery for ACC treated with mitotane (Lysodren, HRA Pharma, Paris, France) and oral hydrocortisone (six males: range 26–66 years, median 50 years and 11 females: range 20–76 years, median 47 years (six of premenopausal age)) and patients receiving hydrocortisone only after surgery in the period before mitotane was started (three males: 23, 54 and 65 years and three females: 37, 39 and 62 years). When there was more than one observation per patient, the median value was used. The mitotane dose was adjusted to maintain therapeutic concentrations of 14–20 mg/l and the hydrocortisone dose ranged from 20 to 50 mg/day. The data listed in Table 1 were obtained from patients in whom plasma mitotane concentrations had reached the therapeutic range and who showed no evidence of ACC recurrence. In six patients, recurrence of ACC took place later in the monitoring period. All patients showed steroid profiles consistent with ACC before surgery, which was confirmed by histology of the removed mass (manuscript in preparation).

Other patient groups chosen for comparison had 5ARD2 deficiency (15 males: range 12–67 years, median 16 years and one female: 7 years) or were treated with finasteride, a specific inhibitor of 5ARD2 (14) (five males: range 42–77 years, median 42 years and one female: 40 years), or with dutasteride, an inhibitor of both 5ARD1 and 5ARD2 (three males: 66, 69 and 77 years).

The control group comprised healthy volunteers (20 males: range 22–49 years, median 31 years and 20 females: range 20–59 years, median 29 years).

### Analytical procedures

Urinary steroid profiling was carried out according to our published method (15). In brief, steroids were extracted and conjugates were hydrolysed enzymatically using *Helix pomatia* digestive juice. The free steroid products were then re-extracted and methyl oxime–trimethylsilyl ether (MO–TMS) derivatives were prepared before analysis by gas chromatography–mass spectrometry (GC–MS) using a Perkin Elmer Clarus 500 system with an OV-1 column (Perkin Elmer, Beaconsfield, Buckinghamshire, UK). Quantification was based on data obtained in cyclic scan mode. Additional procedures were carried out using published protocols for steroid conjugate separation on Sephadex LH-20 (15), solvolysis (16) and sodium borohydride reduction (17). Steroid ratios were compared using one-way ANOVA after logarithmic transformation followed by *post hoc* comparisons by the method of Bonferroni.

## Results

**Figure 2:**
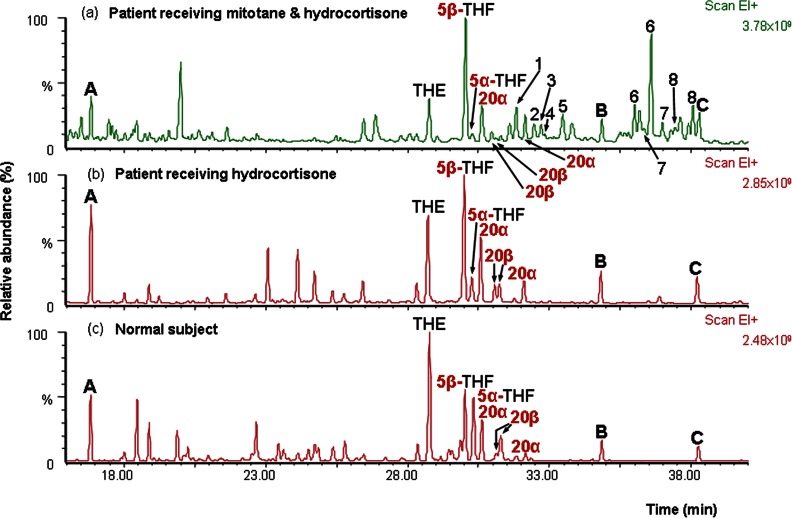
Total ion current chromatograms from GC–MS analysis of urinary steroid metabolites excreted (a) by a patient after surgery when receiving mitotane and hydrocortisone, (b) the same patient on a previous occasion when receiving hydrocortisone only and (c) a normal subject. A, B, C, internal standards: androstanediol, stigmasterol and cholesterol butyrate; THE, tetrahydrocortisone; THF, tetrahydrocortisol (5α- and 5β-reduced epimers shown); 20α- and 20β-, 20-reduced steroids in the order α-cortolone, β-cortol, β-cortolone, α-cortol. Numbered peaks (repeat numbers show syn/anti-pairs of the methyl oxime derived from a single steroid) are 1, 6α-hydroxytetrahydrocortisol; 2, 6α-hydroxy-α-cortolone; 3, 1β-hydroxy-α-cortolone; 4, 6α-hydroxy-β-cortolone; 5, 1β-hydroxy-β-cortolone; 6, 6β-hydroxycortisol; 7, 6β-hydroxy-20β-dihydrocortisol and 8, 6β-hydroxy-20α-dihydrocortisol. Samples were prepared without a wash step (see Results section) to provide good recovery of polar cortisol metabolites. The steroids marked were identified by comparison of mass spectra and retention times with those of authentic standards, or as detailed in the Results section.

Figure 1 summarises the overall effects of mitotane on cortisol catabolism as a background to the findings described in detail below. Figure 2 shows examples of single urinary steroid profiles obtained by GC–MS to illustrate differences between post-surgery ACC patients receiving hydrocortisone with and without mitotane and healthy volunteers. Table 1 lists the cortisol metabolites identified, together with quantitative data based on analyses in all patients.

### 1β- and 6-Hydroxylation

It was evident that there were large increases in polar (late-eluting) cortisol metabolites after mitotane. These were mostly 6-hydroxylated, but smaller amounts of 1β-hydroxylated metabolites also became detectable. None of these were initially apparent during analysis by our standard method, which involves a wash step in which the sample is partitioned between water and ethyl acetate. Omitting this step led to much higher recoveries, showing that the steroids were being lost in the water phase and confirming early observations (10) that a large proportion of cortisol metabolites is found in a polar fraction after mitotane treatment.

Following chromatography of the urine extract on Sephadex LH-20, the polar metabolites were all found in a ‘free plus glucuronide’ fraction (15). None were found in a sulphate fraction. In case there were sulphates conjugated at positions that were resistant to *Helix pomatia* hydrolysis, a chemical cleavage procedure, solvolysis, was also carried out on this fraction. This yielded no additional steroid products. Formation of MO–TMS derivatives of urine extracts before and after *Helix pomatia* hydrolysis gave similar yields of the polar metabolites, showing that nearly all were present as free steroids.

**Figure 3:**
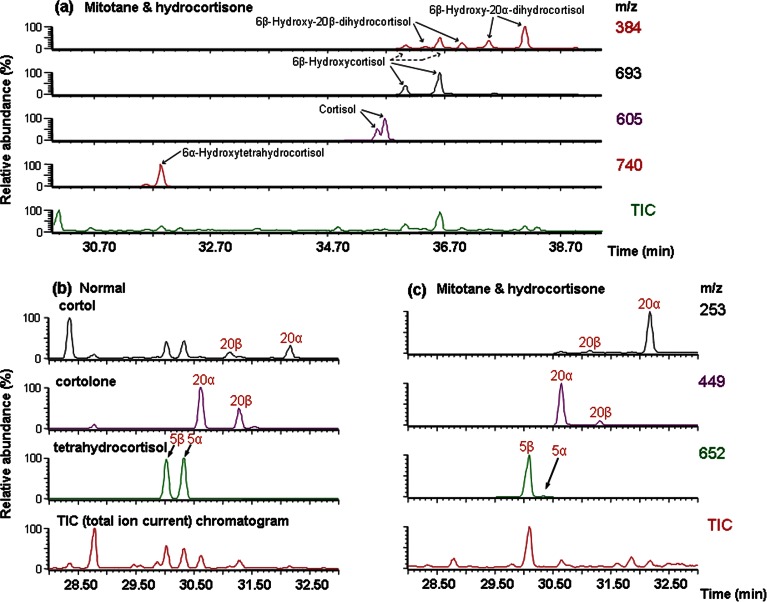
Selected ion chromatograms from GC–MS analysis of urine from a patient receiving mitotane and hydrocortisone in comparison with an untreated subject. Plots of selected ions (*m/z*) and total ion current (TIC) shown are for (a) cortisol and its major 6-hydroxylated metabolites in the patient (where two peaks are marked, these are the syn/anti-pairs of the methyloximes of a single steroid), (b) 5α- and 5β- and 20α- and 20β-reduced pairs of cortisol metabolites for the untreated subject and (c) the same ions for the patient.

The largest component was 6β-hydroxycortisol. Four polar steroid peaks gave similar mass spectra, interpreted as being 6β-hydroxy-20-dihydrocortisols. Their retention times corresponded to 20α- and 20β- epimers, with each giving rise to *syn*/*anti* pairs of the 3-MO. Identification was confirmed by their sodium borohydride reduction products being identical with those obtained by reduction of standard 6β-hydroxycortisol. Small amounts of A-ring-reduced metabolites included 6α-hydroxytetrahydrocortisol, identified by comparison with published data (18) and 1β- and 6α-hydroxycortolones, which matched steroids we have identified in newborns (19). In order to examine the extent of 1β- and 6-hydroxylation a subgroup of samples was analysed without the wash step (Table 1, lower panel). For these, 52% (range 35–52) of total recovered cortisol metabolites were 1β or 6-hydroxylated compared with <2% in controls. Figure 3a shows detection by selected ion monitoring MS of cortisol and its major polar products.

### 5α-Reduction

**Figure 4:**
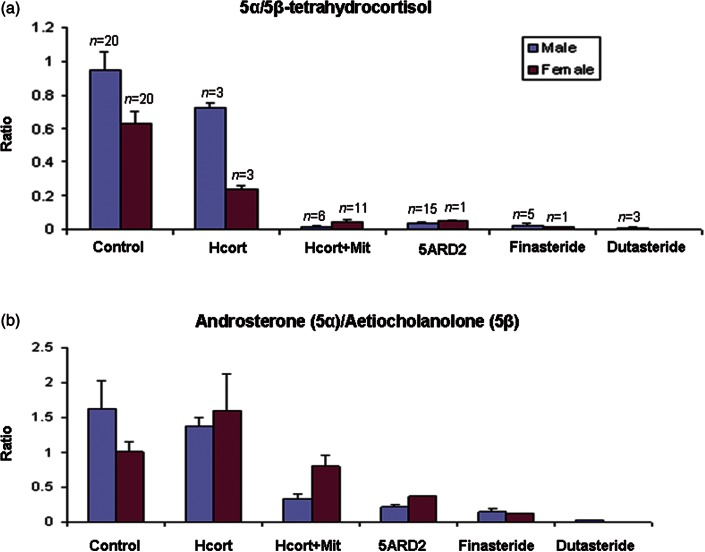
Ratios of 5α/5β-reduced pairs of metabolites of (a) cortisol and (b) androstenedione for females and males in six patient groups. Numbers in each group are shown in (a); bars are for means, with standard errors marked.

A profound decrease in 5α-reduced steroid metabolites relative to their 5β-reduced counterparts was seen in patients receiving mitotane (Figs 2, 3b and c). Figure 4a shows the ratios for 5α/5β-tetrahydrocortisol for controls and five patient groups. These are divided between males and females to take account of known gender differences (20). Hydrocortisone treatment was associated with lower values, as expected (21) (but not significant), while for the mitotane and hydrocortisone group, there was a marked fall in the ratio compared with control (*P*<0.001 (both genders, data for separate genders given in the following paragraphs if different) and hydrocortisone-only groups (*P*<0.001)). There were no significant differences between the mitotane and hydrocortisone group and the remaining groups.

It was noticeable (Fig. 4b) that for the androstenedione metabolites androsterone (5α-reduced) and aetiocholanolone (5β-reduced), the degree of decrease in 5α reduction was not as profound as for cortisol. This mirrors findings in 5ARD2 deficiency (22). There was no change following hydrocortisone treatment, while for the mitotane and hydrocortisone group, there was a fall in the ratio compared with control in males (*P*<0.001) but not in females. Indeed, after mitotane, nine females individually showed an increase in the ratio of androsterone/aetiocholanolone relative to their pre-mitotane values, with single pre- and post-menopausal patients showing falls. In contrast, in all males (*n*=6), the ratio decreased after mitotane was started. There were no significant differences between the mitotane and hydrocortisone group and the remaining groups except for dutasteride, which is known to inhibit both 5ARD1 and 5ARD2, where the ratio was lower (*P*<0.001, compared in males only). This leads to the conclusion that the remaining generation of androsterone relies on the activity of 5ARD1, in contrast to 5α-tetrahydrocortisol generation, which entirely depends on 5ARD2. Mitotane thus appears to specifically inhibit 5ARD2.

All female patients of premenopausal age showed markedly low urinary excretion of androsterone (μg/24 h, mean 111, range 27–255) and aetiocholanolone (mean 165, range 16–222) in comparison with the control group (androsterone (mean 746, range 335–1483, *P*=0.002) and aetiocholanolone (mean 865, range 350–1620, *P*=0.002)). Changes in serum progesterone and urinary pregnanediol, quantified on several occasions in two of the women of reproductive age, indicated that both were cycling. One reported regular bleeding and the other had previously had a hysterectomy. In contrast, male patients showed decrease in androsterone but not in aetiocholanolone.

### 20β-Reduction

**Figure 5:**
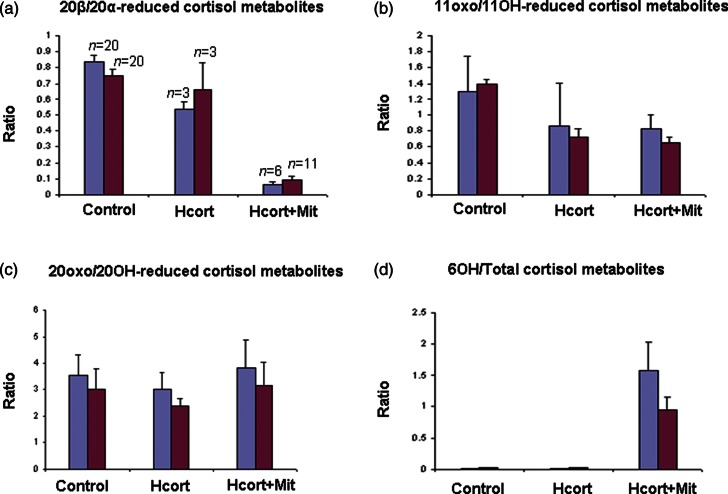
Ratios of cortisol metabolites other than 5α/5β-tetrahydrocortisol for groups of ACC patients and controls. These were calculated from (a) β-cortol+β-cortolone/α-cortol+α-cortolone, (b) tetrahydrocortisone+α- and β-cortolones/5α- and 5β-tetrahydrocortisols+α- and β-cortols, (c) tetrahydrocortisone+5α- and 5β-tetrahydrocortisols/α- and β-cortolones+α- and β-cortols and (d) 6α-hydroxytetrahydrocortisol+6β-hydroxycortisol+6β-hydroxy-20α- and 20β-dihydrocortisol/total cortisol metabolites. Numbers in each group are as shown in (a); bars are for means, with standard errors marked.

A decrease in the 20β-reduced cortisol metabolites relative to their 20α-reduced counterparts was seen in patients receiving mitotane and hydrocortisone (*P*<0.001, Figs 2, 3b and c, 5a). This was most profound for cortols, cortolones and 6α-hydroxycortolones and less marked for 6β-hydroxy-20-dihydrocortisols and 1β-hydroxycortolones (Table 1).

### Other cortisol metabolism

Figure 5b, c and d shows cortisol metabolite ratios that express the effects of mitotane other than on 5α- and 20β-reduction. Figure 5b and c shows that there are no net changes in oxidoreduction at C11 and C20, respectively, indicating that there are no suppressive effects on 11- or 20-hydroxysteroid dehydrogenase activities. Figure 5d shows that increased excretion of 6β-hydroxy steroids occurs only in the hydrocortisone and mitotane group.

### Effect on ACC markers

All patients who had had a recurrence of ACC during mitotane treatment showed steroid excesses before surgery, which included secretion of 11-deoxycortisol, with 20α- and 20β epimers of hexahydro-11-deoxycortisols being excreted in similar amounts. On recurrence, the 20β-reduced epimer was much diminished, as expected. Other major markers showed unchanged metabolism. These were (manuscript in preparation) pregnanediol (20α), pregnanetriol (20α), DHEA, DHEA metabolites, tetrahydro-11-deoxycortisol (5β) and 5-pregnene-3β,16α,20α-triol. This was also expected, as none of these steroids are 5α- or 20β-reduced.

### Dose–response

**Figure 6:**
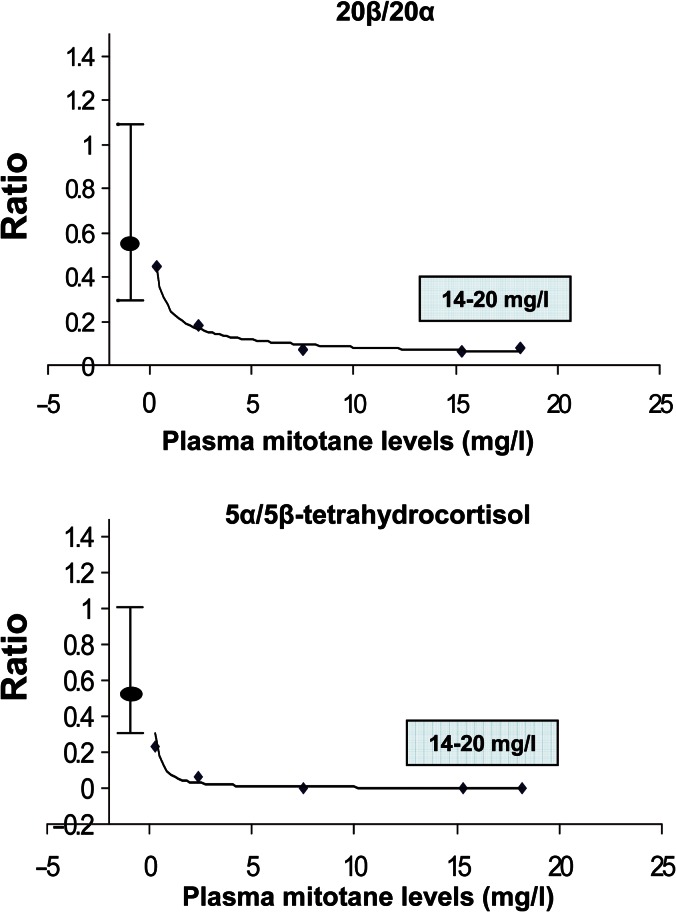
Dose–response relationship for 5α/5β- and 20β/20α-reduced pairs of steroids for one female patient during and at intervals after receiving mitotane. The therapeutic range for mitotane (box) and normal data for the steroid ratios (mean, range I–•–I) are shown. The ratio 20β/20α is derived from the sum of β-cortol+β-cortolone/α-cortol+α-cortolone.

No metabolic changes were found to correlate with either dose or blood concentrations of mitotane when these were within the therapeutic range. However, when blood mitotane was monitored at intervals in a single female patient during an 18 month period following withdrawal of treatment due to neurological complications, a dose–response relationship for inhibition of 5α- and 20β-reduction was evident (Fig. 6). It can be seen that complete inhibition is present for both enzymes at a threshold plasma mitotane concentration much lower than the therapeutic range, and this would explain the lack of correlation noted earlier. The dose response for 6β-hydroxylation was unfortunately not obtained because no material remained for analysis by the method modified to recover very polar steroids. However, in a few patients from whom single samples were taken before plasma mitotane concentrations had attained the therapeutic range, all steroid metabolite ratios were as low as they were in later samples (data not shown).

## Discussion

This study shows that mitotane treatment in patients with ACC results in consistent, but mostly previously unrecognised, changes in cortisol metabolism. These comprise suppression of 5α- and 20β-reduction, together with induction of 1β- and 6-hydroxylation, as summarised in Fig. 1. On limited data, these changes appear to be essentially complete at plasma concentrations of mitotane that are much lower than the therapeutic range.

The urinary 6-hydroxylated metabolites are confirmed as being present at relatively very high concentrations and in unconjugated form. Rapid improvement in patients with Cushing's syndrome (10, 11) and increased hydrocortisone dose requirement during mitotane therapy (1) may thus be explained by decreased bioavailability of oral cortisol due to rapid conversion by CYP3A4 in the liver, perhaps during a first pass after adsorption from the gut. A second factor may be an increase in corticosteroid binding globulin during mitotane treatment (23).

Induction of CYP3A4 by mitotane needs to be considered when selecting drugs and drug dose levels. Kroiss *et al*. (24) have provided a comprehensive list of those likely to be affected. Oral bioavailabilities of the tyrosine kinase inhibitor sunitinib and the anticonvulsant midazolam are profoundly decreased at a plasma mitotane range of 4.9–17.8 mg/l (25).

Polar 1β- and 6α-hydroxylated cortisol metabolites are important components in urine from newborns (19), but the pathways of their formation have not yet been studied. A close relationship between them (Taylor NF, 2012, unpublished observations) suggests that a single enzyme is responsible for both the additional hydroxylations. This might also be CYP3A4, with saturation of the A-ring directing formation of a 6α-hydroxyl and unsaturation directing formation of the better known 6β-hydroxyl, but induction by mitotane of additional cytochromes is also possible.

Mitotane has a specific cytotoxic effect on the mitochondria of adrenal cortical cells and produces focal degeneration of the reticularis and fasciculata zones and only small changes in the zona glomerulosa (6). However, evidence for decrease in cortisol secretion due to mitotane as a contributor to increased hydrocortisone dose requirement is lacking. In a single patient with secondary Cushing's syndrome, short-term treatment did not diminish cortisol metabolic clearance rate nor secretion rate (10). During long-term administration to six patients with various clinical disorders, cortisol secretion rate was diminished in two, did not change in two and increased in two (11). This might be explained by variable compensation by hypothalamo–pituitary–adrenal axis activation.

In contrast, a larger decrease in androgen metabolites post-mitotane in the females than males in this study may more clearly reflect adrenal damage, as there is a greater contribution of the adrenal cortex to these metabolites in females (around 50 vs 30%) (26) and decreased androgen synthesis in the male may be compensated by LH increase. However, the very low androgen metabolite excretion in the females, even in those of premenopausal age, suggests an additional affect on ovarian androgen secretion. As this arises primarily from the theca cells, it is remarkable that cycling was seen to continue in the two treated women on whom we had information.

Our findings of previously unreported effects on cortisol 5- and 20-reduction rely on resolution by GC–MS of individual cortisol metabolites. Early studies would have missed the decrease in 5α-tetrahydrocortisol because the celite chromatographic method used did not resolve it from tetrahydrocortisone (10), while the 20-reduced cortolones and cortols were not reported. The effect on 5-reduction has recently been reported in abstract form (27), in a study also based on GC–MS analysis.

Our interpretation that mitotane probably specifically inhibits 5ARD2 assumes that decrease in the ratio of 5α/5β-reduced metabolites reflects only decrease in 5α-reductase activity, but both mitotane (28) and finasteride (29) also inhibit 5β-reductase (AKR1D1); we could not locate any reports of an effect of dutasteride. This could contribute to the smaller change in these ratios during mitotane and finasteride than during dutasteride. The increase in the ratio of androsterone/aetiocholanolone post-mitotane in females but not in males represents a numerically small change but may reflect a gender difference in balance of gonadal 5-reductases. A third 5α-reductase has recently been reported in prostate cancer cell lines (30). Inhibition of an unrecognised ovarian 5β-reductase is also possible. This enzyme class is widely distributed in vertebrates (31). In a single female patient with documented AKR1D1 deficiency, there was a decrease in 5β-reduced urinary androgen metabolites to 20% of normal (32) but near absence of 5β-tetrahydrocortisol. This could be taken as evidence for the presence of another, unaffected, 5β-reductase isoform.

The 5α-reductase isoform, 5ARD2, which is required for hepatic cortisol metabolism, is the same as that required for generation of dihydrotestosterone from testosterone in androgen target tissues. This is evident from consistent diagnostic decreases in the ratio of urinary 5α/5β-tetrahydrocortisol in patients with 5ARD2 deficiency (22), although a recent review (33) concluded, surprisingly, that hepatic cortisol reduction used 5ARD1, based on *in vitro* findings. Inhibition of 5ARD2 by mitotane could explain our clinical observations that hypoandrogenic male patients on testosterone replacement tend to remain hypoandrogenic at standard doses when also receiving mitotane. This could be explored by measurement of the serum testosterone:dihydrotestosterone ratio or of serum androsterone glucuronide as a marker of peripheral dihydrotestosterone production, but we have not attempted this. Mitotane increases hepatic production of sex hormone binding globulin (SHBG) (23), so this may be another factor. Trial of the androgen analogue, nandrolone (19-nortestosterone), for androgen replacement would be worthwhile. This steroid is rendered less androgenic by 5α-reduction to dihydronandrolone (34), so would be expected to be made more potent by mitotane.

Variable effects of mitotane on 20β-reduction according to substrate may reflect differential effects on substrate binding by a single oxidoreductase or on several. Steroid reductases frequently have wide specificity. While it has been recognised that both AKR1C1 and AKR1C3 show 20α-reductase activity, no enzymes with 20β-reducing activity have yet been defined to our knowledge, even though this reaction in liver is quantitatively important in corticosteroid catabolism. Oxidoreductases for C5 and C20 positions are potentially important as regulators of neurosteroid formation. Mitotane offers the first specific probe for 20β-reduction activity.

It may be concluded that these studies support and expand previous findings, emphasising that mitotane has profound effects on metabolism of cortisol of exogenous origin, and this would diminish oral bioavailability and further activation. The effects on steroid metabolism take place at concentrations that are below the therapeutic range and so could not provide a surrogate for plasma mitotane assay for monitoring capacity for adrenocortical tumour suppression. However, a finding of the characteristic steroid profile changes in patients in whom plasma mitotane concentrations are low, such as when they have only recently commenced mitotane or have ceased treatment and clearance is continuing, might provide a useful reminder that effects on drug dynamics are still likely.

Mitotane in its bioactivated form has potential as a tool for the study of enzymes involved in steroid activation and catabolism, including 20β-reduction activity, for which no specific probes have been previously recognised.

## Author contribution statement

Part of this work has been presented in abstract form (35, 36). Mrs L Ghataore carried out most of the analytical work, wrote text and prepared figures, Dr I Chakraborti reviewed and collated clinical and mitotane concentration data, Dr S Aylwin gave permission for study of patients under his care and provided clinical insights, Mr K-M Schulte gave permission for study of patients under his care on whom he had performed surgery and provided clinical insights, Dr D Dworakowska reviewed and collated clinical and mitotane concentration data and provided clinical insights, Mrs P Coskeran recorded mitotane concentrations and other data relating to nursing care during patient visits and Dr N F Taylor carried out some of the analytical work, wrote text and prepared figures.

## Figures and Tables

**Table 1:** Excretion of metabolites of administered cortisol (each expressed as percentages to take account of variable dosing) in patients receiving hydrocortisone plus mitotane in comparison with excretion of metabolites of endogenous cortisol in controls. The upper panel shows common cortisol metabolites, with values (mean, range) expressed as percentages of the total for these metabolites. The lower panel shows excretion of polar cortisol metabolites in a limited number of patients, with percentages of the total of all cortisol metabolites.

Non polar steroids	**Normal**		**Hydrocortisone+mitotane**	
	Percentage of mean (range)		Percentage of mean (range)	
	Male (*n*=20)	Female (*n*=20)	Male (*n*=6)	Female (*n*=11)
Tetrahydrocortisone	39 (28–55)	39 (28–45)	35 (30–38)	28 (18–40)
5β-Tetrahydrocortisol	17 (11–25)	18 (12–24)	43 (37–53)	45 (35–56)
5α-Tetrahydrocortisol	15 (6–27)	11 (5–26)	0.6 (0.1–0.8)	1.7 (0.1–7)
β-cortolone	6.9 (7–8)	7.3 (4–11)	0.9 (0.2–1.6)	1.5 (1–4)
β-cortol	2.3 (2–3)	2.4 (1–4)	0.3 (0.1–0.5)	0.5 (0.2–1.2)
α-cortolone	8.7 (6–2)	11 (8–16)	14 (9–16)	14 (9–19)
α-cortol	2.6 (1–4)	2.6 (1–4)	6.0 (4–8)	9.2 (4–19)
Polar steroids	Male (*n*=20)	Female (*n*=20)	Male (*n*=4)	Female (*n*=4)
6β-Hydroxycortisol	<2	<2	32 (30–39)	28 (12–37)
6β-Hydroxy-20α-dihydrocortisol	<2	<2	10 (7–17)	10 (3–18)
6β-Hydroxy-20β-dihydrocortisol	<2	<2	4.1 (2–8)	3.6 (1–8)
6α-Hydroxytetrahydrocortisol	<2	<2	5.5 (3–8)	4.9 (3–6)
6α-Hydroxy-α-cortolone	<2	<2	1.6 (1.3–2.2)	1.5 (0.2–2.7)
6α-Hydroxy-β-cortolone	<2	<2	0.1 (0–0.3)	0.06 (0–0.1)
1β-Hydroxy-α-cortolone	<2	<2	1.7 (0.9–3.1)	2.3 (0.8–3.3)
1β-Hydroxy-β-cortolone	<2	<2	1.6 (0.9–2.6)	2.4 (0.6–4.4)

## References

[1] Fassnacht M, Allolio B (2009). Clinical management of adrenocortical carcinoma. Best Practice & Research. Clinical Endocrinology & Metabolism.

[2] Bergenstal DM, Lipsett MB, Moy RH, Hertz R (1959). Regression of adrenal cancer and suppression of adrenal function in men by o,p'DDD. Transactions of the Association of American Physicians.

[3] Terzolo M, Angeli A, Fassnacht M, Daffara F, Tauchmanova L, Conton PA, Rossetto R, Buci L, Sperone P, Grossrubatscher E, Reimondo G, Bollito E, Papotti M, Saeger W, Hahner S, Koschker AC, Arvat E, Ambrosi B, Loli P, Lombardi G, Mannelli M, Bruzzi P, Mantero F, Allolio B, Dogliotti L, Berruti A (2007). Adjuvant mitotane treatment for adrenocortical carcinoma. New England Journal of Medicine.

[4] Martz F, Straw JA (1980). Metabolism and covalent binding of 1-(*o*-chlorophenyl)-1-(*p*-chlorophenyl)-2,2-dichloroethane (o,p-DDD). Drug Metabolism and Disposition.

[5] Fang VS (1979). Cytotoxic activity of 1-(*o*-chlorophenyl)-1-(*p*-chlorophenyl)-2,2-dichloroethane (mitotane) and its analogs on feminizing adrenal neoplastic cells in culture. Cancer Research.

[6] Hart MM, Regan RL, Adamson RH (1973). The effects of isomers of DDD on the ACTH induced steroid output, histology and ultrastructure of the dog adrenal cortex. Toxicology and Applied Pharmacology.

[7] Schteingart DE (2000). Conventional and novel strategies in the treatment of adrenocortical cancer. Brazilian Journal of Medical and Biological Research.

[8] Young RB, Bryson MJ, Sweat ML, Street JC (1973). Complexing of DDT and o,p'DDD with adrenal cytochrome P-450 hydroxylating systems. Journal of Steroid Biochemistry.

[9] Touitou Y, Bogdan A, Luton JP (1978). Changes in corticosteroid synthesis of the human adrenal cortex *in vitro*, induced by treatment with o,p'DDD for Cushing's syndrome: evidence for the future sites of action of the drug. Journal of Steroid Biochemistry.

[10] Southren AL, Tochimoto S, Isurugi K, Gordon GG, Krikun E, Stypulkowski W (1966). The effect of 2,2-bis(2-chlorophenyl-4-chlorophenul)-1,1-dichloroethane (o,p'-DDD) on the metabolism of infused cortisol-7-3H. Steroids.

[11] Fukushima DK, Bradlow H, Hallman L (1971). Effects of o,p'-DDD on cortisol and 6b-hydroxycortisol secretion and metabolism in man. Journal of Clinical Endocrinology.

[12] Hague RV, May W, Cullen DR (1989). Hepatic microsomal enzyme induction and adrenal crisis due to o,p'DDD therapy for metastatic adrenocortical carcinomas. Clinical Endocrinology.

[13] Bledsoe T, Island DP, Ney RL, Liddle GW (1964). An effect of o,p'-DDD on the extra-adrenal metabolism of cortisol in man. Journal of Clinical Endocrinology.

[14] Imperato-McGinley J, Shackleton C, Orlic S, Stoner E (1990). C19 and C21 5β/α metabolite ratios in subjects treated with the 5α-reductase inhibitor finasteride: comparison of male pseudohermaphrodites with inherited 5α-reductase deficiency. Journal of Clinical Endocrinology and Metabolism.

[15] Taylor NF (2006). Urine steroid profiling. In Methods in Molecular Biology, 1, Volume 324, Hormone Assays in Biological Fluids.

[16] Khan SR, Taylor NF (1987). Improved solvolysis of steroid sulphates. Annals of Clinical Biochemistry Supplement.

[17] Christakoudi S, Cowan DA, Taylor NF (2010). Steroids excreted in urine by neonates with 21-hydroxylase deficiency: characterization, using GC–MS and GC–MS/MS, of the D-ring and side chain structure of pregnanes and pregnenes. Steroids.

[18] Setchell KDR, Axelson M, Sjovall J, Kirk DN, Morgan RE (1978). The identification of 6a-hydroxy-tetrahydrocortisol (3α, 6α, 11β, 17α, 21-pentahydroxy-5β-pregnan-20-one) in urine. FEBS Letters.

[19] Taylor NF, Curnow DH, Shackleton CHL (1978). Analysis of glucocorticoid metabolites in the neonatal period: catabolism of cortisone acetate by an infant with 21-hydroxylase deficiency. Clinica Chimica Acta.

[20] Raven PW, Taylor NF (1996). Sex differences in the human metabolism of cortisol. Endocrine Research.

[21] Vanluchene E, Vandekerckhove D, Thiery M, Van Holder R (1981). Changes in cortisol metabolism in various physiological and pathological situations. Annales d'Endocrinologie.

[22] Imperato-McGinley J, Peterson RE, Gautier T, Arthur A, Shackleton C (1985). Decreased urinary C19 and C21 steroid 5α-metabolites in parents of male pseudohermaphrodites with 5α-reductase deficiency: detection of carriers. Journal of Clinical Endocrinology and Metabolism.

[23] Nader N, Raverot G, Emptoz-Bonneton A, Dechaud H, Bonnay EB, Pugeat M (2006). Mitotane has an estrogenic effect on sex hormone binding globulin and corticosteroid and binding globulin in humans. Journal of Clinical Endocrinology and Metabolism.

[24] Kroiss M, Quinkler M, Lutz WK, Allolio B, Fasnacht M (2011). Drug interactions with mitotane by induction of CYP3A4 metabolism in the clinical management of adrenocortical carcinoma. Clinical Endocrinology.

[25] van Erp NP, Guchelaar HJ, Ploeger B, Romijn JA, den Hartigh J, Gelderblom H (2011). Mitotane has a strong and durable inducing effect on CYP3A4 activity. European Journal of Endocrinology.

[26] Azziz R, Nestler JE, Dewailly D. Androgen excess disorders in women. In *Polycystic Ovary Syndrome and other disorders*. 2nd edition. 2006. Humana Press: New Jersey, NJ, USA.

[27] Chortis V, Schneider P, Taylor AE, Tomlinson JW, Hughes BA, Smith DJ, Libe R, Allolio B, Bertagna X, Bertherat J (2011). Steroid profiling in adrenocortical carcinoma reveals mitotane as a strong inducer of CYP3A4 and inhibitor of 5α-reductase with major implications for cortisol and androgen metabolism. Endocrine Reviews.

[28] Ojima M, Saitoh M, Itoh N, Kusang Y, Fukuchi S, Naganuma H (1985). The effects of o'p-DDD on adrenal steroidogenesis and hepatic steroid metabolism. Nihon Naibunpi Gakkai Zasshi.

[29] Drury JE, Di Costanzo L, Penning TM, Christianson DW (2009). Inhibition of human steroid 5β-reductase (AKR1D1) by finasteride and structure of the enzyme–inhibitor complex. Journal of Biological Chemistry.

[30] Uemura M, Tamura K, Chung S, Honma S, Okuyama A, Nakamura Y, Nakagawa H (2008). Novel 5α-steroid reductase (SRD5A3, type-3) is overexpressed in hormone-refractory prostate cancer. Cancer Science.

[31] Langlois VS, Zhang D, Cooke GM, Trudeau VL (2010). Evolution of steroid 5α-reductases and comparison of their function with 5β-reductase. General and Comparative Endocrinology.

[32] Palermo M, Marazzi MS, Hughes BA, Stewart PM, Shackleton CHL (2008). Human delta 4-3-oxosteroid 5β-reductase (AKR1D1) deficiency and steroids metabolism. Steroids.

[33] Miller WL, Auchus RJ (2011). The molecular biology, biochemistry, and physiology of human steroidogenesis and its disorders. Endocrine Reviews.

[34] Toth M, Zakar T (1982). Different binding of testosterone, 19-nortestosterone and their 5α-reduced derivatives to the androgen receptor of the rat seminal vesicle: a step toward the understanding of the anabolic action of nortestosterone. Endokrinologie.

[35] Ghataore L, Abraha H, Chakraborti I, Taylor N, Aylwin S, Schulte K-M (2010). Mitotane treatment has profound effects on cortisol catabolism. Endocrine Abstracts.

[36] Ghataore L, Chakraborti I, Aylwin S, Schulte K-M, Taylor N (2012). Effects of mitotane on exogenous and endogenous steroid metabolism. Endocrine Abstracts.

